# Difficult airway management resources and capnography use in Japanese intensive care units: a nationwide cross-sectional study

**DOI:** 10.1007/s00540-016-2176-3

**Published:** 2016-04-29

**Authors:** Yuko Ono, Koichi Tanigawa, Kazuaki Shinohara, Tetsuhiro Yano, Kotaro Sorimachi, Lubna Sato, Ryota Inokuchi, Jiro Shimada, Choichiro Tase

**Affiliations:** Emergency and Critical Care Medical Center, Fukushima Medical University Hospital, 1 Hikarigaoka, Fukushima, 960-1295 Japan; Fukushima Medical University, Fukushima, Japan; Fukushima Global Medical Science Center, Fukushima, Japan; Department of Anesthesiology, Ohta General Hospital Foundation, Ohta Nishinouchi Hospital, Koriyama, Japan; Department of General and Emergency Medicine, JR Tokyo General Hospital, Tokyo, Japan

**Keywords:** Airway equipment, Capnometry, Supraglottic airway device, Portable storage unit, Postal survey

## Abstract

**Purpose:**

The availability of difficult airway management (DAM) resources and the extent of capnometry use in Japanese intensive care units (ICUs) remained unclear. The purpose of this study was to clarify whether: (1) DAM resources were adequate, and (2) capnometry was routinely applied in Japanese ICUs.

**Methods:**

This nationwide cross-sectional study was conducted from September 2015 to February 2016. All ICUs received a mailed questionnaire about their DAM resources and use of capnometry. Outcome measures were availability of: (1) 24-h in-house backup coverage; (2) a supraglottic airway device (SGA); (3) a dedicated DAM cart; and (4) surgical airway devices, and (5) routine use of capnometry to verify tube placement and for continuous monitoring of ventilator-dependent patients. The association between these outcomes and ICU type (academic, high-volume, closed, surgical) was also analyzed.

**Results:**

Of the 289 ICUs, 196 (67.8 %) returned completed questionnaires. In-house backup coverage and surgical airway devices were highly available (89.3 and 95.9 %), but SGAs and dedicated DAM carts were not (60.2 and 60.7 %). The routine use of capnometry to confirm tube placement was reported by 55.6 % of the ICUs and was highest in closed ICUs (67.2 %, *p* = 0.03). The rate of continuous capnography monitoring was also 55.6 % and was highest in academic ICUs (64.5 %, *p* = 0.04).

**Conclusion:**

In Japanese ICUs, SGAs and dedicated DAM carts were less available, and capnometry was not universally applied either to confirm tube placement, or for continuous monitoring of ventilated patients. Our study revealed areas in need of improvement.

**Electronic supplementary material:**

The online version of this article (doi:10.1007/s00540-016-2176-3) contains supplementary material, which is available to authorized users.

## Introduction

The number of critical care beds in Japan has been growing rapidly. According to data from the Japanese Ministry of Health, Labour and Welfare, the number of intensive care unit (ICU) beds has increased by approximately 50 % in the past several years (http://www.mhlw.go.jp/file/05-Shingikai-12404000-Hokenkyoku-Iryouka/0000101005.pdf; and http://www.mhlw.go.jp/shingi/2009/03/dl/s0325-9k.pdf both in Japanese, accessed 3 March 2016). With the steep growth in critical care, endotracheal intubation (ETI) in ICUs has become much more common. However, ETI in critically ill patients is a challenging procedure because these patients have very little physiological reserve. Severe ETI-related complications, including hypoxia, esophageal intubation, aspiration, and cardiac arrest, are likely to occur in ICUs [1–8], especially when associated with difficult airway management (DAM) [2–8]. The rate of difficult ETI in ICU settings ranges from 10 to 21 % [2–6], which is much higher than the rate in the operating room (OR) [5, 9]. Because ETI-related complications in ICUs are associated with devastating outcomes [8], it has become even more important for ICUs to have proper DAM equipment, and the issue has also become more relevant for intensivists.

Immediate access to appropriate human and equipment resources is a vital element of DAM [8, 10–13]. The limited availability of proper DAM resources are in part responsible for severe ETI-related complications in the ICU [8, 13–15], and many authors strongly recommended that DAM resources in the ICU be the same as those used in the OR [8, 13–15]. Airway management algorithms that have been advocated by the Japanese Society of Anesthesiologists (JSA) [10], the American Society of Anesthesiologists (ASA) [11], and by the Difficult Airway Society (DAS) [12], specify the standard DAM resources for the OR. We previously audited whether Japanese helicopter emergency medical services were adequately equipped regarding these recommended guidelines [16]. However, to date no objective information has been available regarding whether human and equipment resources in Japanese ICUs are compatible with established OR standards [10–12].

Verification of endotracheal tube placement is an indispensable part of any DAM strategy [10–12]. Capnometry is both more sensitive and more specific than auscultation alone in recognizing correct tube placement following emergency intubation [17–19]. In the management of ventilator-dependent patients, early recognition of endotracheal tube dislodgement and obstruction is vital, because loss of airway patency can lead to catastrophic consequences [8, 14]. Therefore, numerous authors, including a national survey performed in the UK, have clearly recommended continuous capnography monitoring from intubation to extubation in the ICU [8, 13, 14], as in the OR. However, the current use of capnography both for verifying endotracheal tube placement and for continuous monitoring of ventilated patients in ICUs in Japan remains unclear.

Therefore, in this study we investigated: (1) the availability of DAM equipment, and specialist care providers; (2) whether these resources are sufficient regarding the JSA, ASA, and DAS guidelines [10–12]; and (3) the current status regarding the use of capnometry for intubation and for continuous capnography monitoring of ventilated patients in Japanese ICUs.

## Materials and methods

### Study design and sites

This nationwide cross-sectional study was conducted from September 2015 to February 2016. After approval by the institutional review boards of Fukushima Medical University (No. 2521), self-administered questionnaires were mailed to the directors of all ICUs (289 hospitals in 47 prefectures) registered as certified training facilities by the Japanese Society of Intensive Care Medicine (JSICM) in November, 2015. A complete list of these hospitals is available at: http://www.jsicm.org/senmon/sisetu_all.html (in Japanese, accessed 7 February 2016). The criteria for a JSICM-certified ICU include: (1) an independent, central clinical division of the facility; (2) one or more dedicated JSICM board-certified intensivists on staff; and (3) more than four critical care beds (http://www.jsicm.org/pdf/senmon_sinsaisoku2016.pdf, in Japanese, accessed 24 March 2016). JSICM-certified ICUs constitute approximately half of all critical care beds in Japan.

### Survey items

When selecting items in the questionnaire, we referred to previous studies conducted in other countries and addressing both similar settings (ICUs [20–24], ORs [25–28], and emergency departments [29–31],) and different settings (pre-hospital emergency medical services [32–34], and obstetrical units [35–37]).

An English version of the Japanese questionnaire used in this study is available in the supplementary material (Online Resource 1). Survey items consisted of basic information regarding the numbers of hospital beds, ICU beds, annual ICU admissions in 2014, the types of ICU, and the availability of the following materials in the ICU: (1) direct laryngoscope and adjunct equipment (curved blade, straight blade, McCoy laryngoscope, stylet, gum elastic bougie, tube exchanger catheter, and local anesthetic spray); (2) alternate intubation equipment (rigid video laryngoscope, flexible fiberscope, retrograde intubation kit, and surgical airway equipment); (3) alternate ventilation equipment [supraglottic airway device (SGA), and oral and nasal airways]; (4) capnometry; (5) a portable packaged unit containing several DAM kits (DAM cart); and (6) neuromuscular blocking agents to facilitate endotracheal intubation and reversal agents (sugammadex, neostigmine, flumazenil, and naloxone). In this survey, we divided ICU types into (a) academic or community, (b) closed or not-closed, (c) high-volume or not, (d) emergency or surgical or other type including medical, mixed, and pediatric ICUs. Academic ICUs were defined as units in university-affiliated hospitals [38]. Closed ICUs were defined as units that transferred all patients to an intensive care team that directs patients’ care with primary responsibility for the therapeutic plan and patient care [39, 40]. Non-closed ICUs were defined as ICUs where the intensive care team provides expertise via elective or mandatory consultation without primary responsibility for the patient care [39, 40]. High-volume ICUs were defined as units in the upper tertile of annual patient admissions [38], and emergency ICUs were defined as units in which most patients were from an emergency room, and which were likely to receive patients suffering acute-onset medical conditions, or surgical illnesses including trauma, burns, intoxication, acute coronary disease, and stroke. We included coronary care units and stroke care units in the emergency category. Surgical ICUs were defined as units in which most patients were from ORs, and which were likely to provide post-operative intensive care.

The questionnaire also asked about the availability of direct laryngoscopes and alternate ventilation equipment in assorted sizes; the product name of the rigid video laryngoscopes and SGA used was also requested. Surgical airway equipment was categorized as a cricothyroidotomy kit, or a set containing a scalpel and hemostat. If capnometry was available, we asked whether: (a) capnometry was used to verify tube placement (routinely, sometimes, and never) [20], and (b) whether the ICU used continuous capnography monitoring for ventilator-dependent patients (routinely, sometimes, and never) [20]. If a dedicated DAM cart was present in the ICU, we asked respondents to specify the contents. We also requested information on: the usual number of on-duty staff ICU physician(s) during the day and overnight; whether in-house experienced (anesthetic or emergency medicine) back-up coverage can be called during overnight hours; and whether staff physicians were board-certified. We included senior residents (post-graduate year 3 or more) as staff ICU physicians, but not junior residents (post-graduate year 1 or 2). We deemed that “24-h in-house back-up coverage” was obtainable if: (a) two or more physicians were usually on duty, including overnight, or (b) in-house experienced back-up coverage was available overnight. Board-certified physicians were defined based on the Japanese Medical Specialty Board criteria (http://www.japan-senmon-i.jp/, in Japanese, accessed 7 February 2016). ICUs that did not respond to the initial survey were sent a repeat mailing on January 2016.

### Outcome measures

Outcomes of interest in this study were availability of: (1) 24-h in-house back-up coverage, (2) an SGA, (3) a DAM cart, (4) surgical airway equipment; and routine use of: (5) capnometry to confirm ETI, and (6) continuous capnography monitoring of ventilator-dependent patients. Of these, (1)–(4) are important DAM resources commonly endorsed by the JSA, ASA, and DAS airway management guidelines [10–12]. The availability of “surgical airway equipment” was defined as a cricothyroidotomy kit or a scalpel and hemostat, present in the ICU.

### Statistical analysis

First, all survey items were evaluated using descriptive statistics. Second, the association between our outcomes of interest (DAM resources and the use of capnometry), and ICU type (academic, closed, high-volume, and surgical) were analyzed using Fisher’s exact test. For this statistical evaluation, we excluded missing data and used complete data sets. All statistical analyses were performed using IBM SPSS Statistics for Windows, version 21.0 (IBM Corp., Armonk, NY, USA), and *p* < 0.05 was considered statistically significant.

### Sample size

During the planning of this study, we performed a power analysis using G*Power 3 for Windows (Heinrich Heine University, Düsseldorf, Germany). Because no previous study, to our knowledge, has examined the association between the type of ICU and DAM resources, we assumed an effect size using Cohen’s power table (Power primer) [41]. With an effect size, “w” of 0.3 (medium size [41]), 88 samples per group (total, 176) provided 80 % power at two-tailed *α* of 0.05.

## Results

Of the 289 Japanese ICUs, 196 returned a completed questionnaire (response rate, 67.8 %). Table 1 shows the demographic information of the responding ICUs. The median number of annual ICU admissions was 688 (interquartile range 530–1000, upper tertile 878); the median number of ICU beds was 10 (interquartile range 6–12). Of these, 47.4 % were academic ICUs, 33.9 % were closed, 29.5 % were surgical, and 34.7 % were emergency units.

**Table 1:** Demographic data of 196 Japanese intensive care units (ICUs)

Basic information	*N* (inter-quartile range)
Hospital beds	613 (500–832)
ICU beds	10 (6–12)
Annual ICU admissions	688 (530–1000)

ICU type	*N* (%)
By funding institute (*N* = 196)	
Academic	93 (47.4)
Community	103 (52.6)
By management (*N* = 192)	
Closed	65 (33.9)
Non-closed	127 (66.1)
By patient characteristic (*N* = 193)	
Surgical	57 (29.5)
Emergency	67 (34.7)
Other	69 (35.8)

Based on the replies of 196 of the 289 ICUs queried

Table 2 summarizes the intubation, alternate intubation, and alternate ventilation equipment available in Japanese ICUs. Among the ICUs that responded, a curved laryngoscope blade and stylet were universally available, 118 (60.2 %) had an SGA, and 188 (95.9 %) possessed a surgical airway device; either a cricothyroidotomy kit (84.7 %), or scalpel and hemostat (11.2 %). Dedicated DAM carts were present in 119 (60.7 %) ICUs, but the contents varied; almost all contained rigid laryngoscope blades of variable design and size (92.4 %), and tracheal tubes of assorted sizes (93.3 %) (Table 3). Although the availability of capnometry was high (92.9 %), the percentage of ICUs routinely using capnometry for ETI and continuous monitoring of ventilated patients were both 55.6 % (Table 4).

**Table 2:** Intubation equipment, alternate intubation equipment, and alternate ventilation equipment in 196 Japanese intensive care units (ICUs)

Equipment item	*N* (%)
1. Direct laryngoscope and adjunct equipment^a^	
A. Curved laryngoscope blade (Macintosh type)	196 (100)
Assorted sizes	192 (98.0)
B. Straight laryngoscope blade (Miller type)	93 (47.4)
Assorted sizes	80 (40.8)
C. McCoy laryngoscope	32 (16.3)
D. Stylet	196 (100)
E. Gum elastic bougie	119 (60.7)
F. Tube exchanger catheter	154 (78.6)
G. Local anesthetic spray	156 (79.6)
2. Alternate intubation equipment	
A. Rigid video laryngoscope^a^	165 (84.2)
a. Airway scope^®^	134 (68.4)
b. McGRATH MAC^®^	102 (52.0)
c. GlideScope^®^	11 (5.6)
d. C-MAC^®^	3 (1.5)
e. Airtraq^®^	2 (1.0)
f. King Vision^®^	1 (0.5)
g. MultiViewScope^®^	1 (0.5)
h. COOPDECH Video Laryngoscope^®^	1 (0.5)
B. Flexible fiberscope	182 (92.9)
C. Retrograde intubation kit	11 (5.6)
D. Surgical airway equipment	188 (95.9)
a. Cricothyroidotomy kit	166 (84.7)
b. Scalpel and hemostat	22 (11.2)
3. Alternate ventilation equipment^a^	
A. SGA^a^	118 (60.2)
Assorted sizes	110 (56.1)
a. I-gel^®^	68 (34.7)
b. Air-Q^®^	16 (8.2)
c. LMA ProSeal^®^	40 (20.4)
d. LMA Classic^®^	28 (14.3)
e. LMA Supreme^®^	3 (1.5)
f. LMA Flexible^®^	1 (0.5)
g. Laryngeal tube^®^	2 (1.0)
h. Ambu AuraOnce^®^	2 (1.0)
i. Ambu Aura-i^®^	1 (0.5)
j. Combitube^®^	1 (0.5)
B. Oral airway	183 (93.4)
C. Nasal airway	192 (98.0)

Based on the replies of 196 of the 289 ICUs queried

*SGA* supraglottic airway device

^a^ICUs may have more than one of the specified equipment items

**Table 3:** Portable storage unit (DAM cart) and its contents in 196 Japanese intensive care units (ICUs)

Item	*N* (%)
Portable storage unit (DAM cart)	119 (60.7)

Contents of the DAM cart	*N* = 119
Rigid laryngoscope blades in various designs and sizes	110 (92.4)
Rigid video laryngoscope	85 (71.4)
Tracheal tubes in assorted sizes	111 (93.3)
Magill forceps	94 (79.0)
Gum elastic bougie	57 (47.9)
Tube exchanger catheter	62 (52.1)
SGA	74 (62.2)
Airway (oral/nasal)	105 (88.2)
Surgical airway device	69 (58.0)
Capnometry	36 (30.3)
Sugammadex	16 (13.4)
Bag valve mask	87 (73.1)
Yankauer suction tip	27 (22.7)
Other devices	13 (10.9)

Based on the replies of 196 of the 289 ICUs queried

*DAM* difficult airway management, *SGA* supraglottic airway device

**Table 4:** Frequency of using capnometry for ETI, and continuous capnography monitoring of ventilated patients in 196 Japanese intensive care units (ICUs)

Item	*N* (%)
Capnometry	182 (92.9)
Use of capnometry to confirm ETI	
Routinely	109 (55.6)
Sometimes	51 (26.0)
Never	36 (18.4)
Continuous capnography monitoring of ventilated patients	
Routinely	109 (55.6)
Sometimes	63 (32.1)
Never	24 (12.2)

Based on the replies of 196 of the 289 ICUs queried

*ETI* endotracheal intubation

Table 5 lists the neuromuscular blocking agents available to facilitate ETI in the responding ICUs, and Table 6 provides information on the ICU manpower, and specialty of ICU physicians. Two or more staff intensivists were usually on duty at 138 ICUs (70.4 %) during the day, and 68 ICUs (34.7 %) overnight. In-house skilled back-up coverage (anesthesiology or emergency medicine) was available in 107 ICUs (54.6 %) overnight. According to our feasibility criteria, 24-h in-house back-up staff was available in 175 (89.3 %) ICUs. Among 2546 attending physicians at all ICUs, the most common board certification was emergency medicine (24.9 %), followed by anesthesiology (24.6 %), and intensive care (18.6 %).

**Table 5:** Neuromuscular blocking agents used to facilitate ETI, and reversal agents in 196 Japanese intensive care units (ICUs)

Item	*N* (%)
A. Neuromuscular blocking agents^a^	
a. Rocuronium	167 (85.2)
b. Vecuronium	68 (34.7)
c. Pancuronium	3 (1.5)
d. Succinylcholine	25 (12.8)
e. Other neuromuscular blocking agents	0 (0)
B. Reversal agents^a^	
a. Sugammadex	128 (65.3)
b. Flumazenil	124 (63.3)
c. Naloxone	94 (48.0)
d. Neostigmine	57 (29.1)

Based on the replies of 196 of the 289 ICUs queried

^a^ICUs may have more than one drug

**Table 6:** Number of on-duty intensive care unit (ICU) physicians and their specialty

Item	*N* (%)
Number of on-duty ICU physicians	*N* = 196
A. Day time	
a. One	58 (29.6)
b. Two or more	138 (70.4)
B. Overnight	
a. One	128 (65.3)
b. Two or more	68 (34.7)
c. In-house experienced back-up coverage^a^ available	107 (54.6)
Board certification of ICU physicians^b^	*N* = 2546
a. Anesthesiology	626 (24.6)
b. Emergency medicine	633 (24.9)
c. Intensive care	474 (18.6)
d. General surgery	271 (10.6)
e. Cardiovascular surgery	87 (3.4)
f. Cranial surgery	101 (4.0)
g. Orthopedics	83 (3.3)
h. Cardiovascular medicine	213 (8.4)
i. Respiratory medicine	34 (1.3)
j. Renal medicine	33 (1.3)
k. Pediatrics	101 (4.0)
l. Other board certification	158 (6.2)

Based on the replies of 196 of the 289 ICUs queried

^a^Back-up from anesthesiology or emergency department

^b^Physicians may have more than one board certification

**Fig. 1:**
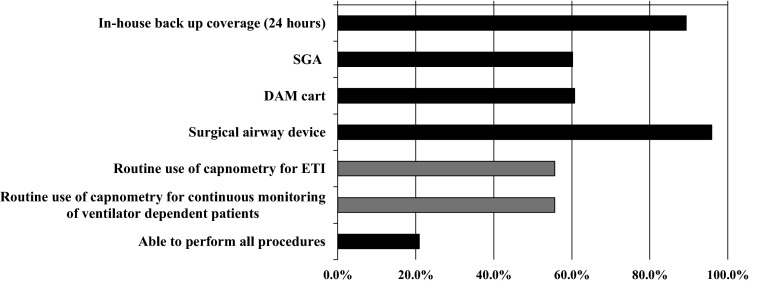
Availability of DAM resources as specified in the JSA, ASA, and DAS guidelines, and routine use of capnometry in Japanese ICUs. *ASA* American Society of Anesthesiologists, *ETI* endotracheal intubation, *DAM* difficult airway management, *DAS* Difficult Airway Society, *ICU* intensive care unit, *JSA* Japanese Society of Anesthesiologists, *SGA* supraglottic airway device

Figure 1 shows the availability of the DAM resources specified in the JSA, ASA, and DAS guidelines [10–12], and routine use of capnometry in Japanese ICUs. According to our feasibility definitions, back-up staff was always deemed available in 89.3 % of ICUs, and surgical airway devices in 95.9 %. The feasibility of the remaining outcomes were all approximately 60 % of ICUs. There were 41 (20.9 %) facilities in which all steps were deemed achievable in the ICUs. Table 7 shows the associations between the feasibility of outcomes of interest and ICU type. There was a general trend that academic, closed, and high-volume ICUs were well-resourced. The rate of routine use of capnometry to confirm ETI was significantly higher in closed ICU [odds ratio 2.0, 95 % confidence interval (CI) 1.1–3.7, *p* = 0.03], but significantly lower in surgical ICUs (odds ratio 0.5, 95 % CI 0.3–0.9, *p* = 0.03). The percentage of routine continuous capnography monitoring of ventilated patients was significantly higher in academic ICUs (odds ratio 1.9, 95 % CI 1.1–3.4, *p* = 0.04). Table 8 compares the availability of DAM resources and the use of capnometry in ICUs in Japan versus other nations.

**Table 7:** Association between the availability of DAM resources and the use of capnometry, and ICU type

Item	*N* (%)	Odds ratio (95 % CI)	*p*
24-h back-up coverage			
Academic ICU, *N* = 93	86 (92.5)	1.9 (0.7–5.0)	0.2
Closed ICU, *N* = 65	59 (90.8)	1.3 (0.5–3.4)	0.8
High-volume ICU, *N* = 63	60 (95.2)	3.1 (0.9–10.3)	0.08
Surgical ICU, *N* = 57	51 (89.5)	1.0 (0.4–2.8)	1.0
Supraglottic airway device			
Academic ICU, *N* = 93	61 (65.6)	1.4 (0.8–2.6)	0.2
Closed ICU, *N* = 64	44 (68.8)	1.6 (0.9–3.1)	0.2
High-volume ICU, *N* = 64	39 (60.9)	1.1 (0.6–2.1)	0.8
Surgical ICU, *N* = 57	38 (66.7)	1.4 (0.7–2.7)	0.3
Surgical airway device			
Academic ICU, *N* = 93	91 (97.8)	3.0 (0.6–14.3)	0.3
Closed ICU, *N* = 65	63 (96.9)	1.5 (0.3–7.7)	1.0
High-volume ICU, *N* = 63	61 (96.8)	1.4 (0.3–7.3)	1.0
Surgical ICU, *N* = 57	56 (98.2)	3.0 (0.4–24.7)	0.4
DAM cart			
Academic ICU, *N* = 93	60 (64.5)	1.3 (0.7–2.3)	0.5
Closed ICU, *N* = 64	44 (68.8)	1.6 (0.8–3.0)	0.2
High-volume ICU, *N* = 62	43 (69.4)	1.6 (0.9–3.1)	0.2
Surgical ICU, *N* = 57	34 (59.6)	0.9 (0.5–1.7)	0.7
Routine use of capnometry to confirm ETI			
Academic ICU, *N* = 93	57 (61.3)	1.5 (0.8–2.6)	0.2
Closed ICU, *N* = 64	43 (67.2)	2.0 (1.1–3.7)	0.03
High-volume ICU, *N* = 62	37 (59.7)	1.2 (0.7–2.2)	0.6
Surgical ICU, *N* = 57	25 (43.9)	0.5 (0.3–0.9)	0.03
Routine continuous capnography monitoring			
Academic ICU, *N* = 93	60 (64.5)	1.9 (1.1–3.4)	0.04
Closed ICU, *N* = 64	41 (64.1)	1.6 (0.9–3.0)	0.2
High-volume ICU, *N* = 62	35 (56.5)	1.0 (0.5–1.8)	1.0
Surgical ICU, *N* = 57	31 (54.4)	0.9 (0.5–1.6)	0.8
Able to perform all procedures			
Academic ICU, *N* = 93	22 (23.7)	1.4 (0.7–2.7)	0.4
Closed ICU, *N* = 65	18 (27.7)	1.8 (0.9–3.6)	0.1
High-volume ICU, *N* = 63	12 (19.0)	0.8 (0.4–1.8)	0.7
Surgical ICU, *N* = 57	9 (15.8)	0.6 (0.3–1.4)	0.3

*CI* confidence interval, *DAM* difficult airway management, *ETI* endotracheal intubation, *ICU* intensive care unit

**Table 8:** International comparison of available DAM resources and routine use of capnometry in ICUs

Authors	Georgiou et al. [20]	Kannan et al. [21]	Haviv et al. [22]	Cumming et al. [23]	Porhomayon et al. [24]	Present study
Country	UK and the Republic of Ireland	UK	Israel	UK	USA	Japan
Year reported	2010	2003	2012	2005	2010	2016
In-house 24-h back-up coverage (%)	N/R	N/R	N/R	N/R	N/R	89.3
SGA (%)	97.8	N/R	100	N/R	80.0	60.2
DAM cart (%)	94.3	N/R	N/R	N/R	70.0	60.7
Surgical airway device (%)	N/R	N/R	86.0^a^	N/R	38.0^a^	95.9
Routine use of capnometry for ETI (%)	31.7	15.7	N/R	10.5	N/R	55.6
Routine use of capnometry for continuous monitoring of ventilated patients (%)	25.4	N/R	N/R	N/R	N/R	55.6

*DAM* difficult airway management, *ETI* endotracheal intubation, *ICU* intensive care unit, *N/R* not recorded, *SGA* supraglottic airway device

^a^This represents availability of a cricothyroidotomy kit. This does not include availability of scalpels and hemostats

## Discussion

This national survey clarified the currently available DAM resources and extent of capnometry use in Japanese ICUs and revealed the areas in need of improvement. To comply with current recommendations, as specified in JSA [10], ASA [11], and DAS [12] guidelines, the availability of SGAs and dedicated DAM carts in Japanese ICUs must be improved. Capnometry is not universally used to confirm correct tube placement, nor is it being used for continuous monitoring of ventilator-dependent patients. End-tidal carbon dioxide (ETCO_2_) confirmation of tube placement and the continuous monitoring of ventilator-dependent patients are ideal safety management practices.

### Use of capnometry in Japanese intensive care units

Our results showed that only 55.6 % of ICUs routinely use capnometry for ETI verification, and the same percentage always monitor capnography in ventilator-dependent patients. However, this percentage was much higher than in previous studies conducted in other countries (Table 8). Our results suggest that ETCO_2_ monitoring was successfully transferred from ORs to ICUs to a certain extent in Japan, but that there is still room for improvement.

The increased use of capnography in the ICU is the single change with the greatest potential to prevent deaths from airway complications in ICUs and elsewhere, outside the OR [14]. A national audit in the UK [8] found that failure to use capnometry in treating a difficult airway contributed to at least some of the fatal outcomes in ICUs. Jaber et al. [1] recently reported that after the introduction of an “intubation bundle” including the routine use of capnometry, ETI-related complications in critically ill patients were significantly reduced.

Displaced tracheostomy and tracheal tubes were the greatest cause of major morbidity and mortality in ICUs [8, 14]. In fact, failure to use capnography in ventilated patients likely contributed to more than 70 % of ICU deaths [8, 14]. Accordingly, further incorporation of ETCO_2_ confirmation and continuous monitoring in Japanese ICUs would improve patient management by critical care medical staff.

### Neglect of the importance of a supraglottic airway device as a rescue ventilation device in Japanese intensive care units

In this study, an SGA was available in only 60.2 % of Japanese ICUs. In other nations, SGAs are available in 80–100 % of critical care departments (Table 8). Therefore, in Japan, SGAs have been undervalued as rescue ventilation devices in critical care settings. Our previous study found the same trend in the pre-hospital setting [16], which further supports this undervaluation. Each ICU must have back-up ventilation strategies [10–12] because: (1) the consequences of failed intubation, especially in the ICU, can be devastating [8, 14]; and (2) airway difficulties are far more likely in the ICU than in the OR [2–6]. Since the use of SGAs is well supported in rescue ventilation strategies [10–12], the standardization of airway equipment, including SGAs, would be beneficial for Japanese ICUs.

### Presence of a dedicated difficult airway management cart in Japanese intensive care units

Our survey results revealed that a dedicated DAM cart was present in 60.7 % of Japanese ICUs, and that the contents varied considerably. This percentage is smaller than previous reports (Table 8). In an ICU, the availability of a DAM cart may have an even greater impact than in the OR, because areas outside the OR, including critical care departments, may not otherwise have immediate access to equipment for airway management [24]. Generally, time is very limited in airway management of a critically ill patient, because these patients have very little physiological reserve. Therefore, every ICU should have immediate access to at least one DAM cart [8, 10–12], which should have the same contents and layout as those used in that hospital’s OR [8]. Suggestions for the contents of DAM carts are in the JSA [10], ASA [11], and DAS [12] guidelines.

### The association between the type of intensive care unit, and the availability of difficult airway management resources and the use of capnometry

This study found a general trend showing that high-volume, closed, and academic ICUs had well-prepared DAM equipment. We also noted that capnometry was more likely to be used for ETI verification in closed ICUs, and continuous capnography monitoring was more likely in academic ICUs. It is well known that these types of ICUs have improved patient outcomes compared with other ICU types [38–40, 42, 43]. Therefore, it is possible, at least in part, that having well-prepared DAM resources is responsible for improved outcomes. This fact, and our data, further support current recommendations that every ICU have DAM resources at the same level as that of hospital ORs [8, 13–15]. We also observed that surgical ICUs were less likely to use capnometry for ETI verification, but we could not explain the reason for this finding, based on our survey results.

### Study limitations and advantages

There are four major limitations to this study. First, we did not include non-JSICM certified ICUs, which comprise another 50 % of all critical-care beds in Japan. This is because a complete list of non-JSICM certified ICUs was not available. However, it is likely that DAM resources are less available and capnometry is used less often in non-JSICM-certified training facilities, because most such ICUs are not academic or closed units. Our recommendations regarding DAM resources and the use of capnometry can also be applied to non-JSICM-certified ICUs. Second, our survey did not determine the frequencies of difficult airways situations (i.e., cannot ventilate, cannot intubate), nor did our survey obtain information on the clinical protocols for DAM in Japanese ICUs. Third, because our questionnaire was self-administered, reporting bias is possible. Fourth, we did not clarify why SGAs and DAM carts were less available, nor why capnometry was under-used in Japanese ICUs. These points require further investigation. Despite these limitations, this study has several strengths. First, the response rate was quite high (196 of 289 surveyed ICUs responded), and our survey successfully captured the findings in various types of ICU from all geographic areas in Japan including closed, surgical, emergency, and other types. Our study provides an accurate depiction of the current state of advanced airway management in Japanese ICUs. Second, our findings provided the associations between ICU type, and DAM resources and the use of capnometry. To the best of our knowledge, this relationship has not been clarified previously. We believe that this study is a meaningful first approach to improve DAM in Japanese ICUs.

In conclusion, this nationwide cross-sectional study clarified the available DAM equipment in Japanese ICUs, as well as areas that warrant improving. Available best evidence clearly states that DAM resources in ICUs should be consistent with those in the OR [8, 13–15], and the use of capnometry should meet the same standards that apply in the hospital’s OR [8, 13–15]. Therefore, it would be helpful for many Japanese ICUs to standardize DAM equipment, including an SGA and a DAM cart, and to incorporate the routine use of capnometry into their clinical practices.

## Electronic supplementary material

Below is the link to the electronic supplementary material.

Supplementary material 1 (DOCX 40 kb)
