# Supplementary material for: Difficult airway management resources and capnography use in Japanese intensive care units: a nationwide cross-sectional study
Source: J Anesth. 2016 Apr 29;30:644–52. doi: 10.1007/s00540-016-2176-3 (PMC4956707; doi:10.1007/s00540-016-2176-3)
Supplement: Supplementary file 1 — Supplementary material 1 (DOCX 40 kb) [file 540_2016_2176_MOESM1_ESM.docx]

| **Airway management equipment survey in Japanese intensive care units: A questionnaire** | | | | | | | | | | | | | | | | | | | | | | | | | | | | | | | | | | | | | | | | | | | | | | | | | | | | | | | | | | | | |
| --- | --- | --- | --- | --- | --- | --- | --- | --- | --- | --- | --- | --- | --- | --- | --- | --- | --- | --- | --- | --- | --- | --- | --- | --- | --- | --- | --- | --- | --- | --- | --- | --- | --- | --- | --- | --- | --- | --- | --- | --- | --- | --- | --- | --- | --- | --- | --- | --- | --- | --- | --- | --- | --- | --- | --- | --- | --- | --- | --- | --- |
|  |  |  |  |  |  |  |  |  |  |  | |  |  |  |  |  | |  |  |  | |  | |  | |  | |  | |  | |  | |  | |  | |  | |  | |  | |  | |  | |  | |  | |  | |  | | |  | | |  |
|  |  |  | **Note**  Please fill out this form based on the situation in your hospital in November 2015, unless any annotations are provided. | | | | | | | | | | | | | | | | | | | | | | | | | | | | | | | | | | | | | | | | | | | | | | | | | | | | |  | | |  | |
|  |  |  |  |  |  |  |  |  |  |  |  |  |  |  |  |  |  |  |  |  |  |  |  |  |  |  |  |  |  |  |  |  |  |  |  |  |  |  |  |  |  |  |  |  |  |  |  |  |  |  |  |  |  |  |  |  | | |  | |
|  |  |  |  |  |  |  |  |  |  |  |  |  |  |  |  |  |  |  |  |  |  |  |  |  |  |  |  |  |  |  |  |  |  |  |  |  |  |  |  |  |  |  |  |  |  |  |  |  |  |  |  |  |  |  |  |  | | |  | |
|  |  |  |  |  |  |  |  |  |  |  | |  |  |  |  |  | |  |  |  | |  | |  | |  | |  | |  | |  | |  | |  | |  | |  | |  | |  | |  | |  | |  | |  | |  | | |  | | |  |
| **Hospital name** | | | | | | | | | | | | | | | | | | | | | | |  | | | | | | | | | | | | | | | | | | | | | | | | | | | | | | | | | | | | | |
| **1．How many beds does your hospital have?** | | | | | | | | | | | | | | | | | | | | | | |  | | | | | | | | | | | | | | | | | | | | | | | | | | | | | | | | | | | | | |
| **2．How many beds does your intensive care unit (ICU) have?** | | | | | | | | | | | | | | | | | | | | | | |  | | | | | | | | | | | | | | | | | | | | | | | | | | | | | | | | | | | | | |
| **3．How many patients did your ICU receive in 2014?** | | | | | | | | | | | | | | | | | | | | | | |  | | | | | | | | | | | | | | | | | | | | | | | | | | | | | | | | | | | | | |
| **4. Which best describes the type of your ICU?** | | | | | | | | | | | | | | | | | | | | | | | | | | | | | | | | | | | | | | | | | | | | | | | | | | | | | | | | | | | | |
| (1) Please choose one: | | | | | | | | | | | | | | | | | | | | | | |  | |  | |  | |  | |  | |  | |  | |  | |  | |  | |  | |  | |  | |  | |  | |  | |  | | |  | | |
| □ Closed ICU:  (An intensive care team directs patient care and takes primary responsibility for the therapeutic plan and patient care.)  □ Non closed ICU (An intensive care team provides expertise without primary responsibility for the patient care) | | | | | | | | | | | | | | | | | | | | | | | | | | | | | | | | | | | | | | | | | | | | | | | | | | | | | | | | | | | | |
| (2) Please choose one: | | | | | | | | | | | | | | | | | | | | | | |  | |  | |  | |  | |  | |  | |  | |  | |  | |  | |  | |  | |  | |  | |  | |  | |  | | |  | | |
| □ Surgical ICU: Most patients are from the operating room  □ Emergency ICU: Most patients are from the emergency department (Please include CCU and Stroke Care Units)  □ Other: (e.g., Medical ICU, Mixed ICU, and Pediatric ICU) | | | | | | | | | | | | | | | | | | | | | | | | | | | | | | | | | | | | | | | | | | | | | | | | | | | | | | | | | | | | |
| **5. Is the following airway equipment available in your ICU?** | | | | | | | | | | | | | | | | | | | | | | | | | | | | | | | | | | | | | | | | | | | | | | | | | | | | | | | | | | | | |
| 1. **Direct laryngoscope and adjunct equipment** | | | | | | | | | | | | | | | | | | | | | | |  | |  | |  | |  | |  | |  | |  | |  | |  | |  | |  | |  | |  | |  | |  | |  | |  | | |  | | |
| A. Curved laryngoscope blade (Macintosh type)  (a) assorted sizes | | | | | | | | | | | | | | | | | | | | | | | | | | | **□ Yes □ No**  **□ Yes □ No** | | | | | | | | | | | | | | | | | | | | | | | | | | | | | | | | | |
| B. Straight laryngoscope blade (Miller type)  (a) Assorted sizes | | | | | | | | | | | | | | | | | | | | | | | | | | | **□ Yes □ No**  **□ Yes □ No** | | | | | | | | | | | | | | | | | | | | | | | | | | | | | | | | | |
| C. McCoy laryngoscope | | | | | | | | | | | | | | | | | | | | | | | | | | | **□ Yes □ No** | | | | | | | | | | | | | | | | | | | | | | | | | | | | | | | | | |
| D. Stylet | | | | | | | | | | | | | | | | | | | | | | | | | | | **□ Yes □ No** | | | | | | | | | | | | | | | | | | | | | | | | | | | | | | | | | |
| E. Gum elastic bougie | | | | | | | | | | | | | | | | | | | | | | | | | | | **□ Yes □ No** | | | | | | | | | | | | | | | | | | | | | | | | | | | | | | | | | |
| F. Tube exchanger catheter | | | | | | | | | | | | | | | | | | | | | | | | | | | **□ Yes □ No** | | | | | | | | | | | | | | | | | | | | | | | | | | | | | | | | | |
| G. Local anesthetic spray | | | | | | | | | | | | | | | | | | | | | | | | | | | **□ Yes □ No** | | | | | | | | | | | | | | | | | | | | | | | | | | | | | | | | | |
| (2) **Alternative intubation equipment** | | | | | | | | | | | | | | | | | | | | |  | |  | |  | |  | |  | |  | |  | |  | |  | |  | |  | |  | |  | |  | |  | |  | |  | |  | | |  | | |
| A. Rigid video laryngoscope | | | | | | | | | | | | | | | | | | | | | | | | | | | **□ Yes □ No** | | | | | | | | | | | | | | | | | | | | | | | | | | | | | | | | | |
| B. If yes, please provide the product name.  Note: If you have more than one rigid video laryngoscope, please enumerate. | | | | | | | | | | | | | | | | | | | | | | | | | | |  | | | | | | | | | | | | | | | | | | | | | | | | | | | | | | | | | |
| C. Flexible fiberscope | | | | | | | | | | | | | | | | | | | | | | | | | | | **□ Yes □ No** | | | | | | | | | | | | | | | | | | | | | | | | | | | | | | | | | |
| D. Retrograde intubation kit | | | | | | | | | | | | | | | | | | | | | | | | | | | **□ Yes □ No** | | | | | | | | | | | | | | | | | | | | | | | | | | | | | | | | | |
| (3) **Alternative ventilation equipment** | | | | | | | | | | | | | | | | | | | | | | | | | | |  | |  | |  | |  | |  | |  | |  | |  | |  | |  | |  | |  | |  | |  | |  | | |  | | |
| A. Supraglottic airway device | | | | | | | | | | | | | | | | | | | | | | | | | | | **□ Yes □ No**  **□ Yes □ No** | | | | | | | | | | | | | | | | | | | | | | | | | | | | | | | | | |
| (a) Assorted sizes | | | | | | | | | | | | | | | | | | | | | | | | | | |  |  |  |  |  |  |  |  |  |  |  |  |  |  |  |  |  |  |  |  |  |  |  |  |  |  |  |  |  |  |  |  |  |  |
| B. If yes, please provide the product name.  Note: If you have more than one supraglottic airway, please enumerate. | | | | | | | | | | | | | | | | | | | | | | | | | | |  | | | | | | | | | | | | | | | | | | | | | | | | | | | | | | | | | |
| C. Oral airway | | | | | | | | | | | | | | | | | | | | | | | | | | | **□ Yes □ No** | | | | | | | | | | | | | | | | | | | | | | | | | | | | | | | | | |
| D. Nasal airway | | | | | | | | | | | | | | | | | | | | | | | | | | | **□ Yes □ No** | | | | | | | | | | | | | | | | | | | | | | | | | | | | | | | | | |
| (4) **Surgical airway device** | | | | | | | | | | | | | | | | | | | | | | | | | | |  | |  | |  | |  | |  | |  | |  | |  | |  | |  | |  | |  | |  | |  | |  | | |  | | |
| A. Cricothyroidotomy kit | | | | | | | | | | | | | | | | | | | | | | | | | | | **□ Yes □ No**  **□ Only scalpel and hemostat** | | | | | | | | | | | | | | | | | | | | | | | | | | | | | | | | | |
| (5) **Device to confirm endotracheal intubation** | | | | | | | | | | | | | | | | | | | | | | | | | | |  | |  | |  | |  | |  | |  | |  | |  | |  | |  | |  | |  | |  | |  | |  | | |  | | |
| A. Capnometry (EtCO_2_ monitor) | | | | | | | | | | | | | | | | | | | | | | | | | | | **□ Yes □ No** | | | | | | | | | | | | | | | | | | | | | | | | | | | | | | | | | |
| B. If yes, please answer the following question: | | | | | | | | | | | | | | | | | | | | | | | | | | |  | |  | |  | |  | |  | |  | |  | |  | |  | |  | |  | |  | |  | |  | |  | | |  | | |
| (a) Does your ICU team use capnometry to confirm correct tube placement? | | | | | | | | | | | | | | | | | | | | | | | | | | | **□ Routinely □ Sometimes**  **□ Never** | | | | | | | | | | | | | | | | | | | | | | | | | | | | | | | | | |
| (a) Does your ICU team continuously monitor capnography for ventilator-dependent patients? | | | | | | | | | | | | | | | | | | | | | | | | | | | **□ Routinely □ Sometimes**  **□ Never** | | | | | | | | | | | | | | | | | | | | | | | | | | | | | | | | | |
| (6) **Drugs** | | | | | | | | | | | | | | | | | | | | | | | | | | |  | |  | |  | |  | |  | |  | |  | |  | |  | |  | |  | |  | |  | |  | |  | | |  | | |
| Are the following drugs available in your ICU? (Please select all that apply.) | | | | | | | | | | | | | | | | | | | | | | | | | | | | | | | | | | | | | | | | | | | | | | | | | | | | | | | | | | | | |
| A. Neuromuscular blocking agents | | | | | | | | | | | | | | | | | | | | | | | | | | | | | | | | | | | | | | | | | | | | | | | | | | | | | | | | | | | | |
| □ Rocuronium □ Vecuronium □ Pancuronium □ Succinylcholine  □ Other neuromuscular blocking agents | | | | | | | | | | | | | | | | | | | | | | | | | | | | | | | | | | | | | | | | | | | | | | | | | | | | | | | | | | | | |
| B. Reversal agents | | | | | | | | | | | | | | | | | | | | | | | | | | | | | | | | | | | | | | | | | | | | | | | | | | | | | | | | | | | | |
| □ Sugammadex □ Naloxone □ Flumazenil □ Neostigmine  □ Other reversal agents | | | | | | | | | | | | | | | | | | | | | | | | | | | | | | | | | | | | | | | | | | | | | | | | | | | | | | | | | | | | |
|  | | | | | | | | | | | | | | | | | | | | | | | | | | |  | |  | |  | |  | |  | |  | |  | |  | |  | |  | |  | |  | |  | |  | |  | | |  | | |
| (7) **Portable packaged unit containing difficult airway management (DAM) equipment** | | | | | | | | | | | | | | | | | | | | | | | | | | | | | | | | | | | | | | | | | | | | | | | | | | | | | | | | | | | | |
| A. Does your ICU have a dedicated portable packaged unit (e.g., DAM cart, DAM bag)? | | | | | | | | | | | | | | | | | | | | | | | | | | | **□ Yes □ No** | | | | | | | | | | | | | | | | | | | | | | | | | | | | | | | | | |
| B. If yes, is the following equipment contained in the portable packaged unit?  (Please select all that apply.) | | | | | | | | | | | | | | | | | | | | | | | | | | | | | | | | | | | | | | | | | | | | | | | | | | | | | | | | | | | | |
| □ Rigid laryngoscope blades in various designs and sizes □ Rigid video laryngoscope  □ Tracheal tubes in assorted sizes □ Magill forceps □ Gum elastic bougie  □ Tube exchanger catheter □ Airway (oral/nasal) □ Supraglottic airway device  □ Surgical airway device □ Capnometry □ Sugammadex  □ Bag valve mask □ Yankauer suction tip | | | | | | | | | | | | | | | | | | | | | | | | | | | | | | | | | | | | | | | | | | | | | | | | | | | | | | | | | | | | |
| □ Other devices (please specify) | | | | | | | | | | | | | | | | |  | | | | | | | | | | | | | | | | | | | | | | | | | | | | | | | | | | | | | | | | | | | |
| **6. ICU physicians** | | | | | | | | | | | | | | | | | | | | | | | | | | | | | | | | | | | | | | | | | | | | | | | | | | | | | | | | | | | | |
| A. How many ICU physicians does your hospital have? (Please include senior residents (post-graduate year 3 or more), but not junior residents (post-graduate year 1 or 2).) | | | | | | | | | | | | | | | | | | | | | | | | | | |  | | | | | | | | | | | | | | | | | | | | | | | | | | | | | | | | | |
| B. Is more than one physician usually on duty during the day in your ICU? | | | | | | | | | | | | | | | | | | | | | | | | | | | **□ Yes □ No** | | | | | | | | | | | | | | | | | | | | | | | | | | | | | | | | | |
| C. Is more than one physician usually on duty overnight in your ICU? | | | | | | | | | | | | | | | | | | | | | | | | | | | **□ Yes □ No** | | | | | | | | | | | | | | | | | | | | | | | | | | | | | | | | | |
| D. If no is applied the above question, can you obtain in-house experienced (anesthetic or emergency medicine) back-up coverage overnight? | | | | | | | | | | | | | | | | | | | | | | | | | | | **□ Yes □ No** | | | | | | | | | | | | | | | | | | | | | | | | | | | | | | | | | |
| **B.** Of these ICU physicians, how many are board-certified in the fields listed below?*  *Physicians may have more than one board certification. | | | | | | | | | | | | | | | | | | | | | | | | | | | | | | | | | | | | | | | | | | | | | | | | | | | | | | | | | | | | |
| Intensive care | | | | | | | | | | |  | | | | | | | | | | | | Anesthesia | | | | | | | | | | | | | | | | | | | |  | | | | | | | | | | | | | | | | | |
| Emergency medicine | | | | | | | | | | |  | | | | | | | | | | | | General surgery | | | | | | | | | | | | | | | | | | | |  | | | | | | | | | | | | | | | | | |
| Cardiovascular surgery | | | | | | | | | | |  | | | | | | | | | | | | Cranial surgery | | | | | | | | | | | | | | | | | | | |  | | | | | | | | | | | | | | | | | |
| Orthopedics | | | | | | | | | | |  | | | | | | | | | | | | Cardiovascular medicine | | | | | | | | | | | | | | | | | | | |  | | | | | | | | | | | | | | | | | |
| Respiratory medicine | | | | | | | | | | |  | | | | | | | | | | | | Renal medicine | | | | | | | | | | | | | | | | | | | |  | | | | | | | | | | | | | | | | | |
| Pediatrics | | | | | | | | | | |  | | | | | | | | | | | | Other board certifications | | | | | | | | | | | | | | | | | | | |  | | | | | | | | | | | | | | | | | |
| **7. Protocol questions** | | | | | | | | | | | | | | | | | | | | | | | | | | | | | | | | | | | | | | | | | | | | | | | | | | | | | | | | | | | | |
| 1. Are there any recommendations or protocols in place when you perform emergency endotracheal intubation in your ICU? | | | | | | | | | | | | | | | | | | | | | | | | | | | | | | | | | | | | | | | | | | | | | | | | | | | | | | | | | | | | |
|  | | | | | | | | | | | | | | | | | | | | | | | | | | | | | | | | | | | | | | | | | | | | | | | | | | | | | | | | | | | | |
| 1. Are there any recommendations or protocols to prevent accidental extubation in your ICU? | | | | | | | | | | | | | | | | | | | | | | | | | | | | | | | | | | | | | | | | | | | | | | | | | | | | | | | | | | | | |
|  | | | | | | | | | | | | | | | | | | | | | | | | | | | | | | | | | | | | | | | | | | | | | | | | | | | | | | | | | | | | |
| 1. Are there any recommendations or protocols in place when you extubate a patient in your ICU? | | | | | | | | | | | | | | | | | | | | | | | | | | | | | | | | | | | | | | | | | | | | | | | | | | | | | | | | | | | | |
|  | | | | | | | | | | | | | | | | | | | | | | | | | | | | | | | | | | | | | | | | | | | | | | | | | | | | | | | | | | | | |
| 1. Other comments | | | | | | | | | | | | | | | | | | | | | | | | | | | | | | | | | | | | | | | | | | | | | | | | | | | | | | | | | | | | |
|  | | | | | | | | | | | | | | | | | | | | | | | | | | | | | | | | | | | | | | | | | | | | | | | | | | | | | | | | | | | | |
|  |  |  |  |  |  |  |  |  |  |  | |  |  |  |  |  | |  |  |  | |  | |  | |  | |  | |  | |  | |  | |  | |  | |  | |  | |  | |  | |  | |  | |  | |  | | |  | | |  |
| **Thank you very much for your time and collaboration.** | | | | | | | | | | | | | | | | | | | | | | | | | | | | | | | | | | | | | | | | | | | | | | | | | | | | | | | | | | | | |
